# Reducing Gadolinium Exposure in Patients Undergoing Monitoring for Meningiomas

**DOI:** 10.7759/cureus.37492

**Published:** 2023-04-12

**Authors:** Srikar Sathraju, Kristen Johnson, Kyle V Cicalese, Charles F Opalak, William C Broaddus

**Affiliations:** 1 Neurosurgery, Virginia Commonwealth University School of Medicine, Richmond, USA; 2 Neurosurgery, Richmond VA Medical Center, Richmond, USA; 3 Neurosurgery, Prisma Health Southeastern Neurosurgical and Spine Institute, Greenville, USA

**Keywords:** inter-rater reliability, mri surveillance, gadolinium deposition illness, gadolinium-based contrast agent, meningioma

## Abstract

Background

Due to the non-malignant and slow-growing nature of many meningiomas, surveillance with serial magnetic resonance imaging (MRI) serves as an acceptable management plan. However, repeated imaging with gold-standard contrast-based studies may lead to contrast-associated adverse effects. Non-gadolinium T2 sequences may serve as a suitable alternative without the risk of adverse effects of contrast. Thus, this study sought to investigate the agreement between post-contrast T1 and non-gadolinium T2 MRI sequences in the measurement of meningioma growth.

Methodology

The Virginia Commonwealth University School of Medicine (VCU SOM) brain tumor database was used to create a cohort of meningioma patients and determine the number of patients who had T1 post-contrast imaging accompanied by readily measurable imaging from either T2 fast spin echo (FSE) or T2 fluid-attenuated inversion recovery (FLAIR) sequences. Measurements of the largest axial and perpendicular diameters of each tumor were conducted by two independent observers using T1 post-contrast, T2 FSE, and T2 FLAIR imaging series. Lin’s concordance correlation coefficient (CCC) was calculated to assess inter-rater reliability between observers and agreement between measurements of tumor diameter among the different imaging sequences.

Results

In total, 33 patients (average age = 72.1 ± 12.9 years, 90% female) with meningiomas were extracted from our database, with 22 (66.7%) undergoing T1 post-contrast imaging accompanied with readily measurable imaging from T2 FSE and/or T2 FLAIR sequences. The inter-rater reliability between the measurements of T1 axial and perpendicular diameters was 0.96 (95% confidence interval (CI) = 0.92-0.98) and 0.92 (95% CI = 0.83-0.97), respectively. The inter-rater reliability between the measurements of T2 axial perpendicular diameters was 0.93 (95% = CI 0.92-0.97) and 0.89 (95% CI = 0.74-0.95), respectively. The agreements between the measurement of T1 and T2 FSE axial diameter by each observer were 0.97 (95% CI = 0.93-0.98) and 0.92 (95% CI = 0.81-0.97). The agreements between the measurements of T1 and T2 FSE perpendicular diameter measurements by each observer were 0.98 (95% CI = 0.95-0.99) and 0.88 (95% CI = 0.73-0.95).

Conclusions

Two-thirds of our patients had meningiomas that were readily measurable on either T2 FSE or T2 FLAIR sequences. Additionally, there was excellent inter-rater reliability between the observers in our study as well as an agreement between individual measurements of T1 post-contrast and T2 FSE tumor diameters. These findings suggest that T2 FSE may serve as a safe and similarly effective surveillance method for the long-term management of meningioma patients.

## Introduction

Meningiomas are the most common primary intracranial tumor with incidence rates of 8.6 per 100,000 persons [1]. Due to their non-malignant and slow-growing nature, surgical intervention is not always a necessity, and surveillance via imaging can be an acceptable management plan for asymptomatic patients [2]. Appropriate time intervals for follow-up are at the discretion of individual physicians [2,3]. Given the potential for long-term follow-up (more than five years) and associated multiple imaging scans with gold-standard T1 post-contrast magnetic resonance imaging (MRI) scans, current practice results in repeated administration of contrast agents [2].

Gadolinium (Gd^3+^) is the chief ingredient of MRI contrast agents, shortening the relaxation time of T1 imaging to produce images that display tissues with greater contrast than those without [4,5]. Following approval of the Food and Drug Administration (FDA) in 1988, gadolinium-based contrast agents (GBCAs) have been widely used for complex clinical diagnosis and are considered extremely safe, with adverse life-threatening reactions occurring at rates of 0.001-0.01% [6].

GBCAs are categorized into linear and macrocyclic subgroups, corresponding to their chelation and structure. Linear agents have been found to be relatively less stable than their counterparts, releasing free Gd^3+^ [4]. In vivo, free Gd^3+^ is highly toxic and can be distributed into adjacent tissues such as the bone, lymph nodes, and liver [4]. Adverse consequences of repeated Gd^3+^ exposure were first reported in a 2006 study which demonstrated that, in patients with impaired renal function, linear GBCAs may contribute to nephrogenic systemic fibrosis (NSF), characterized by the thickening and hardening of the skin at the distal extremities. Subsequently, it has been recommended to use the macrocyclic iteration and limit repeated exposure; however, no limitations have been placed on patients with normal renal function [1,7-9]. In 2014, Kanda and colleagues published the first study which demonstrated Gd^3+^ deposition into brain tissue, specifically with hyperintensity in the dentate nucleus and globus pallidus. These patients had a history of linear GBCA administration but were without renal impairment [10]. Further studies have been conducted with respect to Gd^3+^ deposition into brain tissue but adverse sequelae from Gd^3+^ deposition have not been demonstrated [11].

Although the adverse effects of long-term contrast exposure are rare and their effects on the brain are currently inconclusive, the potential compromise of patient safety warrants novel strategies. In many cases at our institution, previously identified meningiomas have been adequately measured with T2 sequences, suggesting that these patients could potentially forgo the use of repeated gadolinium exposure. Therefore, we set out to determine the percentage of meningioma patients who have been monitored with T2 imaging at our institution as well as compare the extent of agreement of tumor measurements between T1 post-contrast and T2 non-contrast sequences.

## Materials and methods

Study overview

Meningioma patients were extracted from the Virginia Commonwealth University School of Medicine (VCU SOM) brain tumor database, which comprises patients presenting for brain tumors between the years 2005-2015. To create a random sample, patients were placed in alphabetical order using their last name, and every other patient was selected. Spinal meningiomas were excluded from this study. The VCU institutional review board approved our proposal and classified this study as a pilot study as, at the time of approval, we were, to our knowledge, the first to investigate the agreement between measurements of T1 post-contrast and T2 non-contrast meningioma diameters. Thus, we determined, without calculation, that a sample size of roughly 30 patients would be both feasible for our observers and adequate for statistical significance.

Tumor measurement

The Response Evaluation Criteria in Solid Tumors (RECIST) was introduced in 2000 and subsequently updated in 2009. Since its introduction and adoption by the oncological community, RECIST has been widely used to report changes in tumor size [12,13]. Still, the simplified ellipsoid volume (ABC/2) has repeatedly demonstrated adequate correlation with planimetric techniques [14,15]. The measurement techniques employed by the raters are a derivation of the latter.

**Figure 1:**
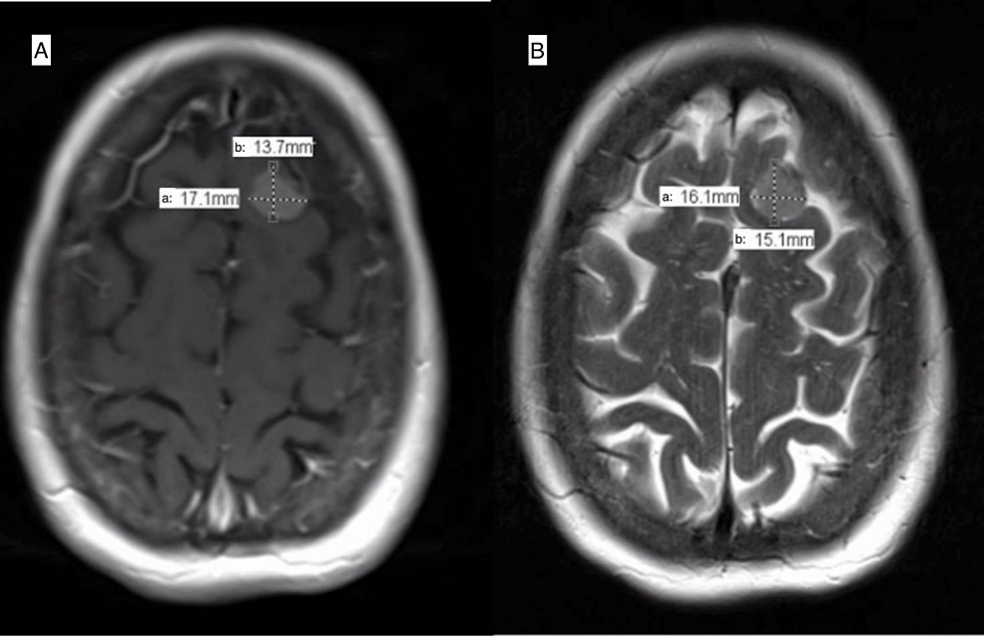
Measuring axial (a) and perpendicular (b) dimension diameters of meningioma T1 post-contrast (A) and T2 FSE (B) images using Philips IntelliSpace Radiology. (A) T1 post-contrast MRI of a meningioma measuring 17.1 mm in the axial (a) dimension and 13.7 mm in its perpendicular (b) dimension; (B) T2 FSE MRI of a meningioma measuring 16.1 mm in the axial (a) dimension and 15.1 mm in its perpendicular (b) dimension. FSE: fast spin echo; MRI: magnetic resonance imaging

Two measurements were taken on each meningioma by two independent observers (or raters), authors Kristen Johnson (KJ) and Srikar N. Sattiraju (SNS), on each axial T1 post-contrast, axial T2 fast spin echo (FSE), and axial T2 fluid-attenuated inversion recovery (FLAIR) image. The first measurement, the longest dimension, was measured and labeled A. The second measurement, B, was the longest perpendicular dimension to A (Figure 1). Both KJ and SNS were trained by the senior author, an experienced surgical neuro-oncologist, on how to appropriately conduct these measurements using conventional imaging software (Philips IntelliSpace Radiology).

Statistical analysis

Demographic and tumor variables including sex, location of the tumor, and patient age at imaging were summarized using means and standard deviations or frequencies and percentages. Lin’s concordance correlation coefficients (CCC) with 95% confidence intervals (CIs) were calculated to assess inter-rater reliability and tumor diameter measurement agreement [16]. Inter-rater reliability, expressed using CCC, was calculated to determine the agreement in the raters’ tumor diameter measurements. Bland-Altman analyses were performed to further illustrate the agreement between inter-rater reliability and tumor diameter measurements [17]. CCC and Bland-Altman statistical analyses were conducted using R Statistical Software (Version 3.6.1, Vienna, Austria). In line with the suggested guidelines, CCC values <0.50 were considered to indicate poor inter-rater reliability or agreement, 0.50-0.75 values were moderate, 0.75-0.90 values were good, and >0.90 values were excellent [18].

## Results

Patient demographics

A total of 739 patients were diagnosed with meningioma at VCU between 2005 and 2015, and 33 of these patients were extracted for this study. The mean age of our patient sample at initial MRI imaging was 72.1 ± 12.9 years, ranging from 49 to 92 years with a median of 75 years. There were 30 (90%) females and three (10%) males. There were 10 (30.3%) patients with meningiomas located in the frontal fossa, the most frequently occurring tumor location within our sample, followed by four (12.1%) sphenoid wing meningiomas. This demographic information is summarized in Table 1.

**Table 1:** Characteristics of 33 patients with meningiomas.

Characteristic	Study cohort (n = 33)
Female	30 (90%)
Age at initial imaging (years)	72.1 ± 12.9
Tumor location	
Frontal lobe	10 (30.3%)
Sphenoid wing	4 (12.1%)
Skull base	3 (9.1%)
Cavernous sinus	2 (6.1%)
Olfactory groove	2 (6.1%)
Sella turcica	2 (6.1%)
Middle fossa	2 (6.1%)
Clinoid process	1 (3.0%)
Frontoparietal	1 (3.0%)
Foramen magnum	1 (3.0%)
Occipital	1 (3.0%)
Parietal	1 (3.0%)
Posterior fossa	1 (3.0%)
Sagittal/parasagittal	1 (3.0%)
Tentorium cerebelli	1 (3.0%)

Inter-rater reliability

Observer 1, SNS, was able to successfully measure 22 meningiomas on either T2 FSE or T2 FLAIR images. Observer 2, KJ, was able to successfully measure 20 meningiomas on either T2 FSE or T2 FLAIR images. Thus, 66.6% of meningiomas could reliably be measured with non-contrasted scans in our cohort. The inter-rater reliability of meningioma diameters between the two observers is shown in Table 2.

**Table 2:** Inter-rater reliability of meningioma diameters between observers KJ and SNS. CCC: Lin’s concordance correlation coefficient; SNS: Srikar N. Sattiraju (rater initials); KJ: Kristen Johnson (rater initials); MRI: magnetic resonance imaging; FSE: fast spin echo; FLAIR: fluid attenuated inversion recovery *: a bias correction factor that measures how far the best-fit line deviates from a line at 45 degrees. No deviation from the 45-degree line occurs when C.b = 1 [16].

Interpreter MRI comparison	Patients (n)	CCC (95% CI)	Scale shift	Location shift	Bias correction factor*
KJ vs. SNS axial (A) T1	29	0.96 (0.92-0.98)	0.92	-0.08	0.99
KJ vs. SNS perpendicular (B) T1	29	0.92 (0.83-0.96)	0.97	-0.05	1.00
KJ vs. SNS axial (A) T2	18	0.93 (0.82-0.97)	0.99	-0.04	1.00
KJ vs. SNS perpendicular (B) T2	18	0.89 (0.74-0.95)	0.98	0.33	0.95
KJ vs. SNS axial (A) FLAIR	19	0.91 (0.79-0.96)	0.97	-0.26	0.97
KJ vs. SNS perpendicular (B) FLAIR	18	0.95 (0.89-0.98)	0.95	-0.01	1.00

**Figure 2:**
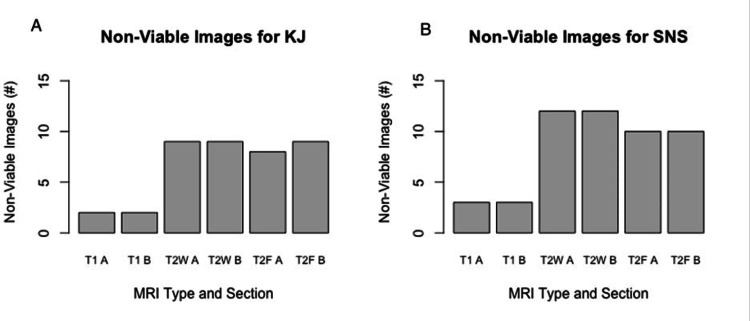
Bar chart depicting the total number of each observer’s non-viable images for each MRI technique and image section. (A) Bar chart representing the number of MRIs by sequence and by dimension (A: axial; B: perpendicular) that could not be readily interpretable by rater KJ; (B) Bar chart representing the number of MRIs by sequence and by dimensions that could not be readily interpretable by rater SNS. T1A: T1 post-contrast MRI in the axial dimension; T1B: T1 post-contrast MRI in the perpendicular dimension; T2W A: T2-weighted FSE MRI in axial dimension; T2W B: T2-weighted FSE MRI in perpendicular dimension;  T2F A: T2 FLAIR MRI image in axial dimension; T2F B: T2 FLAIR MRI image in perpendicular dimension. MRI: magnetic resonance image; KJ: Kristen Johnson (rater initials); SNS: Srikar N. Sattiraju (rater initials); FSE: fast spin echo; FLAIR: fluid-attenuated inversion recovery

KJ and SNS had 29 patients for both T1 axial and perpendicular measurements, 18 patients for both T2 axial and perpendicular measurements, and 19 and 18 patients for T2 FLAIR axial and perpendicular measurements, respectively. The number of non-successful images for each MRI technique per rater is presented in Figure 2.

**Figure 3:**
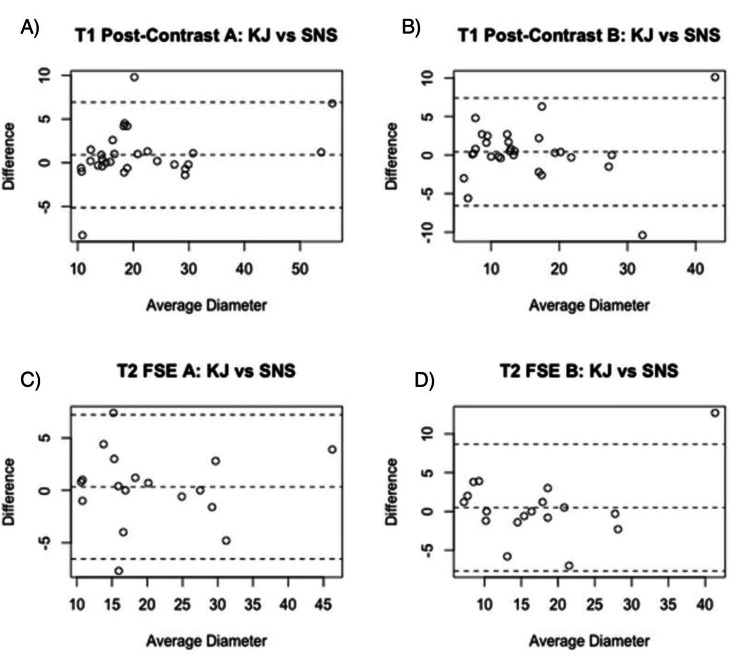
Bland-Altman analyses representing the inter-rater reliability between SNS and KJ among various imaging techniques. (A) Bland-Altman plot representing the inter-rater reliability between SNS and KJ in their measurements of the axial (a) dimension in meningiomas imaged with T1 post-contrast MRI; (B) bland-Altman plot representing the inter-rater reliability between SNS and KJ in their measurements of the perpendicular (b) dimension in meningiomas imaged with T1 post-contrast MRI; (C) Bland-Altman plot representing the inter-rater reliability between SNS and KJ in their measurements of the axial (a) dimension in meningiomas imaged with T2 FSE MRI; (D) Bland-Altman plot representing the inter-rater reliability between SNS and KJ in their measurements of the perpendicular (b) dimension in meningiomas imaged with T2 FSE MRI. SNS: Srikar N. Sattiraju (rater initials); KJ: Kristen Johnson (rater initials); MRI: magnetic resonance imaging; FSE: fast spin echo

The inter-rater reliabilities for these variables were 0.96 (95% CI = 0.92-0.98) for T1 axial; 0.92 (95% CI = 0.83-0.96) for T1 perpendicular; 0.93 (95% CI = 0.82-0.97) for T2 axial; 0.89 (95% CI = 0.74-0.95) for T2 perpendicular; 0.91 (95% CI = 0.79-0.96) for T2 FLAIR axial; and 0.95 (95% CI = 0.89-0.98) for T2 FLAIR perpendicular. Bland-Altman plots illustrating the inter-rater reliability of tumor diameter measurements are presented in Figure 3.

Agreement of tumor diameter measurements between imaging techniques

**Figure 4:**
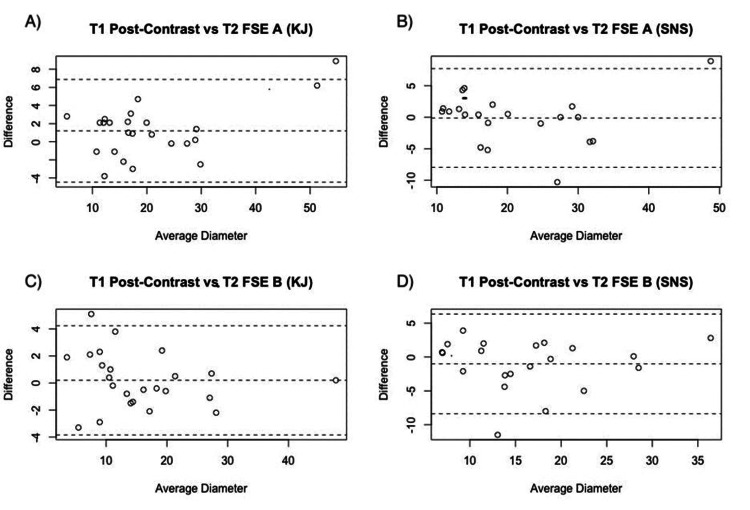
Bland-Altman analyses representing the agreement between each observer’s meningioma diameter measurements using T1 post-contrast and T2 FSE imaging. (A) Bland-Altman plot representing the agreement between rater KJ’s measurements of the axial (a) dimension of meningiomas imaged with both T1 post-contrast and T2 FSE MRI; (B) bland-Altman plot representing the agreement between rater SNS’s measurements of the axial (a) dimension of meningiomas imaged with both T1 post-contrast and T2 FSE MRI; (C) Bland-Altman plot representing the agreement between rater KJ’s measurements of the axial (a) dimension of meningiomas imaged with both T1 post-contrast and T2 FSE MRI; (D) Bland-Altman plot representing the agreement between rater SNS’s measurements of the perpendicular (b) dimension of meningiomas imaged with both T1 post-contrast and T2 FSE MRI. SNS: Srikar N. Sattiraju (rater initials); KJ: Kristen Johnson (rater initials); MRI: magnetic resonance imaging; FSE: fast spin echo

The agreements between measurements of T1 and T2 FSE axial diameters by each rater were 0.97 (95% CI = 0.93-0.98) and 0.92 (95% CI = 0.81-0.97). The agreements between measurements of T1 and T2 FSE perpendicular diameters by each observer were 0.98 (95% CI = 0.95-0.99) and 0.88 (95% CI = 0.73-0.95) (Table 3). Bland-Altman plots illustrating the agreement between individual raters’ tumor diameter measurements are presented in Figure 4.

**Table 3:** Lin’s concordance correlation coefficients of meningioma diameters measured by T1 and T2 MRI scans. CCC: Lin’s concordance correlation coefficient; SNS: Srikar N. Sattiraju (rater initials); KJ: Kristen Johnson (rater initials); MRI: magnetic resonance imaging; FSE: fast spin echo; FLAIR: fluid attenuated inversion recovery *: a bias correction factor that measures how far the best-fit line deviates from a line at 45 degrees. No deviation from the 45-degree line occurs when C.b = 1 [16].

Interpreter MRI Comparison	Patients (n)	CCC (95% CI)	Scale Shift	Location Shift	Bias Correction Factor*
KJ Axial (A) T1 vs T2	24	0.97 (0.93-0.98)	0.89	-0.10	0.99
KJ Perpendicular (B) T1 vs T2	24	0.98 (0.95-0.99)	1.04	-0.02	0.99
KJ Axial T1 vs Flair	25	0.93 (0.85-0.96)	1.17	0.06	0.99
KJ Perpendicular T1 vs Flair	24	0.89 (0.77-0.95)	0.89	0.20	0.97
KJ Axial T2 vs Flair	21	0.95 (0.87-0.98)	1.07	-0.12	0.99
KJ Perpendicular T2 vs Flair	20	0.90 (0.76-0.96)	1.02	0.09	1.00
SNS Axial (A) T1 vs T2	21	0.92 (0.81-0.97)	0.98	0.01	1.00
SNS Perpendicular (B) T1 vs T2	21	0.88 (0.73-0.95)	0.99	0.13	0.99
SNS Axial T1 vs Flair	22	0.92 (0.73-0.97)	0.96	0.18	0.92
SNS Perpendicular T1 vs Flair	22	0.97 (0.94-0.99)	1.09	0.04	1.00
SNS Axial T2 vs Flair	18	0.83 (0.65-0.92)	1.33	0.19	0.94
SNS Perpendicular T2 vs Flair	18	0.80 (0.58-0.92)	1.23	0.24	0.95

The agreements between measurements of T1 and T2 FLAIR axial diameters by each rater were 0.93 (95% CI = 0.85-0.96) and 0.92 (95% CI = 0.73-0.97). The agreements between measurements of T1 and T2 FLAIR perpendicular diameters by each rater were 0.89 (95% CI = 0.77-0.95) and 0.97 (95% CI = 0.94-0.99). The agreements between measurements of T2 FSE and T2 FLAIR axial diameters by each rater were 0.95 (95% CI = 0.87-0.98) and 0.83 (95% CI = 0.65-0.92). The agreements between measurements of T2 FSE and T2 FLAIR perpendicular diameter by each rater were 0.90 (95% CI = 0.76-0.96) and 0.80 (95% CI = 0.58-0.92).

## Discussion

In the present study, there was good and excellent inter-rater reliability between the measurements of meningioma diameters using T1 post-contrast, T2 FSE, and T2 FLAIR images by two similarly trained observers, as indicated by concordance correlation values between 0.75-0.90 and >0.90, respectively, for each type of imaging technique. These findings indicate that the measurements of meningioma diameter obtained from T1 post-contrast, T2 FSE, and T2 FLAIR by these observers are precise and that comparisons of each observer’s measurements between imaging techniques are reflective of the accuracy of the instrument used to measure them. Additionally, a similar study found that two neuroradiologists with 20 years difference in imaging reading were able to maintain moderate agreement between meningioma volume (κ = 0.45), which improved to substantial for meningiomas >1 mL (κ = 0.77), suggesting that even less experienced observers can still make accurate measurements [19]. Because these prerequisite findings demonstrated adequate reliability of our observers and the instruments of measure, subsequent analysis of the agreement between meningioma diameter measurements between imaging techniques was appropriate.

In the present study, there was excellent or near-excellent agreement between both observers’ measurements of axial and perpendicular meningioma diameters when comparing T1 post-contrast and T2 FSE images of individual tumors. These findings are in line with several recent studies comparing the efficacy of T1 and T2 imaging techniques for measuring meningioma growth patterns. For instance, He et al. (2020) found that measurements of the change in tumor size over time for both T2 and T2 FLAIR imaging techniques were significantly correlated with T1+ contrast imaging of 18 asymptomatic meningiomas [20]. Similarly, T2-weighted imaging was able to accurately measure the size and detect changes in meningioma volume with similar efficacy as T1 post-contrast images, especially tumors >1 mL and those located in the posterior fossa [19,21]. Finally, a recent study by Boto and colleagues found excellent agreement, as measured by intraclass correlation coefficients, between various measurements of meningioma size and growth between T1 three-dimensional gadolinium and two-dimensional T2-weighted imaging [22]. Taken together, these findings bolster the evidence that T2 MRI techniques may be similarly efficacious in measuring meningioma size and growth. Unlike the findings presented in the latter three recent reports, we found that only two-thirds of the meningiomas in our cohort could be measured and followed without the administration of gadolinium-based agents. The common factor of the meningiomas that could not be measured accurately in our study was the lack of intensity of the tumors on T2 imaging. Meningiomas of various sizes or locations were able to be measured accurately with T2 imaging by our raters, and thus the limitation of T2 imaging in our study appears to be associated with the MRI characteristics of the tumors themselves as opposed to their location and size.

The fact that the majority of meningiomas can be measured with non-contrast imaging, along with the similar agreement between T1 and T2 measurements of meningioma tumor size and growth in the present study and prior studies could have significant implications for meningioma surveillance. Namely, they provide substantial evidence that a conversion from gold-standard contrast imaging to non-contrast imaging could avoid contrast-related toxicities without sacrificing efficacy in select patients [19-22]. Utilizing non-contrast scans more frequently may also be more cost-effective for patients and the healthcare system [22,23].

Despite these findings, gadolinium-based MRI should remain a critical tool in the management of certain meningioma patients given the superior efficacy of contrast in visualizing various aspects of meningioma growth relative to T2 imaging, including those of smaller volume (i.e., <1 mL), en plaque meningiomas, and those with enhancing dural tails [19,24,25]. Indeed, current European Association of Neuro-Oncology (EANO) guidelines recommend a wait-and-see approach for patients with normal life expectancy who have small (i.e., <3 cm) and/or asymptomatic tumors where contrast imaging is used six months after diagnosis, annually for five years, and then biannually [2]. Our findings reported here suggest that routine imaging with non-contrast MR may serve as a means of providing surveillance for the majority of patients without excessive exposure to gadolinium. We note that about one-third of patients have tumors that cannot be visualized well without contrast. These patients will require post-contrast studies for surveillance imaging. Similarly, following complete resection of meningiomas, contrast will be required to identify small nodules of tumor recurrence.

Limitations

There are several limitations associated with the present study. As the data were collected retrospectively, future research could include prospective or randomized studies of patients who have meningiomas capable of being measured on T2 sequences. Another limitation is our small sample size which may affect generalizability. Multi-institution designs in future studies could help alleviate this limitation. Lastly, the measurements were taken by two observers who were not trained physicians and lacked a formal background in neuroradiology. While future studies may consider using observers with formal neuroradiology training, we believe the findings from this study are important as they show that even informally trained observers can acquire specific neuroradiological diagnostic skills.

## Conclusions

In this study, there was excellent agreement between measurements of T1 post-contrast axial imaging and the T2 FSE imaging in the majority of patients in which their meningiomas could be visualized well with T2 imaging. These findings suggest that non-contrast scans may serve as a safe, reliable, and more cost-effective alternative to gold-standard T1 post-contrast studies in the surveillance of meningiomas in select patients.
